# A *Global Tuberculosis Dictionary:* unified terms and definitions for the field of tuberculosis

**DOI:** 10.1016/S2214-109X(24)00083-4

**Published:** 2024-03-22

**Authors:** Alberto L Garcia-Basteiro, Joanna Ehrlich, Maryline Bonnet, Marianne Calnan, Stephen M Graham, Sabine Hermans, Adriana Jarrett, Rhoda Lewa, Anna Mandalakas, Leonardo Martinez, Giovanni Battista Migliori, Catherine W M Ong, Larissa Otero, Molebogeng Xheedha Rangaka, Mario C B Raviglione, Barbara Seaworth, Willy Ssengooba, Grant Theron, Anete Trajman, Marcel A Behr

**Affiliations:** Centro de Investigação em Saúde de Manhiça, Manhiça 1121, Mozambique; Instituto de Salud Global de Barcelona, Barcelona, Spain; Instituto de Salud Global de Barcelona, Barcelona, Spain; TransVIHMI, University of Montpellier, Institut de Recherche pour le Développement, Institut National de la Santé et de la Recherche Médicale, Montpellier, France; University Research Co., Chevy Chase, MD, USA; University of Melbourne Department of Paediatrics, Royal Children’s Hospital, Melbourne, VIC, Australia; Department of Global Health, Amsterdam Institute for Global Health and Development, Amsterdam UMC, Amsterdam, Netherlands; Department of Infectious Diseases, Centre for Tropical Medicine and Travel Medicine, Amsterdam UMC, Amsterdam, Netherlands; We Are TB, National Tuberculosis Controllers Association, Smyrna, GA, USA; Nairobi, Kenya; Department of Pediatrics, Baylor College of Medicine—Texas Children’s Hospital, Houston, TX, USA; Department of Epidemiology, School of Public Health, Boston University, Boston, MA, USA; Servizio di Epidemiologia Clinica delle Malattie Respiratorie, Istituti Clinici Scientifici Maugeri, IRCCS Tradate, Tradate, Italy; Infectious Diseases Translational Research Programme and Division of Infectious Diseases, Department of Medicine, Yong Loo Lin School of Medicine, National University of Singapore, Singapore; School of Medicine and Instituto de Medicina Tropical Alexander von Humboldt, Universidad Peruana Cayetano Heredia, Lima, Peru; Institute for Global Health & Medical Research Council Clinical Trials Unit, University College London, London, UK; School of Public Health and Clinical Infectious Disease Research Institute-AFRICA, University of Cape Town, Cape Town, South Africa; Centre for Multidisciplinary Research in Health Science, University of Milan, Milan, Italy; University of Texas at Tyler Health Science Center, Tyler, Texas, USA; Department of Medical Microbiology, Makerere University, Kampala, Uganda; DSI-NRF Centre of Excellence for Biomedical Tuberculosis Research, South African Medical Research Council Centre for Tuberculosis Research, Division of Molecular Biology and Human Genetics, Faculty of Medicine and Health Sciences, Stellenbosch University, Cape Town, South Africa; Federal University of Rio de Janeiro, Rio de Janeiro, Brazil; McGill International TB Centre, Montreal, QC, Canada; McGill International TB Centre, Montreal, QC, Canada

Tuberculosis is one of the oldest foes of humankind, with a reach spanning every nation. Tuberculosis has claimed the most lives of any infectious disease in history.^1^ In 2022 alone, there were around 10·6 million disease episodes, and 1·3 million tuberculosis-attributable deaths.^2^ Tuberculosis research holds a prominent position in the scientific landscape, and has one of the most extensive bodies of publications. Scientific outputs in the field of tuberculosis have shown exponential growth since the 1990s,^3^ propelled by renewed investments and unprecedented advances in research, including the development of molecular diagnostics, improved treatment regimens, and a quest for new vaccine candidates.^4–7^

Some of these advances have broadened our understanding of the spectrum of tuberculosis from exposure to infection to disease, challenging long-standing concepts and introducing novel terms for application across research and control efforts. However, the introduction of new, inconsistently-defined terms, or the modification of previous definitions over time, can lead to confusion, misunderstanding, and inconsistency in the use of tuberculosis-related terminology. For instance, the term latent tuberculosis infection has been broadly used to both denote the presence of *Mycobacterium tuberculosis* infection in the body and to define past immunological exposure to *M tuberculosis.* WHO has recently deemed the term inaccurate, and recommends using tuberculosis infection instead.^8^ The term active tuberculosis is frequently used to denote a person with disease caused by *M tuberculosis*, in contrast with the traditional term, latent tuberculosis infection. However, we now know that so-called latent tuberculosis is not truly inactive tuberculosis, or even latent as understood in other fields. Similarly, the widely used case definitions for paediatric tuberculosis (probable tuberculosis, possible tuberculosis, unconfirmed tuberculosis) have undergone multiple revisions by different authors, who use different terms to describe the same condition.^9^ Emerging terms or concepts have garnered substantial attention in recent years, such as incipient tuberculosis and subclinical tuberculosis, yet have been used and defined differently across scientific literature and policy-related documents.^10–12^

In recognition of the dynamic nature of vocabulary and lexicology, particularly in the evolving realm of scientific ventures that aim to challenge established concepts and definitions, there is a need to harmonise and streamline the terminology associated with tuberculosis. The development of a living dictionary could serve as a reference for people interested in tuberculosis, including those engaged in tuberculosis control and research, or those directly affected by this condition. It is hoped that such a resource would contribute to clarity, consistency, and effective communication within the field.

Driven by this motivation, a diverse group of independent researchers, public health officers, and tuberculosis survivors, with different backgrounds, expertise and geographic origins, collaborated to establish the first edition of the Global Tuberculosis Dictionary. We first conducted a comprehensive review of tuberculosis-related literature (published between Jan 1, 2000, and Dec 31, 2022) to identify terms and definitions associated with tuberculosis. All WHO Global Tuberculosis Programme publications were selected as a main source for extraction of terms, as these publications commonly represent an international reference. We also reviewed publications from the International Union Against Tuberculosis and Lung Disease and the US Centers for Disease Control and Prevention. In addition, we conducted a systematic search in PubMed (using the keywords “tuberculosis” AND “definitions” OR “glossary” OR “term”) to identify articles in which the main objective was to discuss definitions of tuberculosis terms and concepts, or those that contained glossaries. Terms were initially screened for their relevance to tuberculosis and were excluded if they were not tuberculosis-specific (eg, “culture” or “adolescent”). Terms were classified into categories and distributed for review by a panel of tuberculosis experts, who were masked to all other reviews and contributed to the dictionary as the editorial team (which includes all authors of this Comment). Each term and definition was assessed by two reviewers from the editorial team, who could reject, amend, or accept each term and definition. Conflicting opinions were solved by consensus or, if needed, by discussion with a third reviewer from the editorial team. All amendments underwent a final review for cohesion and consistency by the editors of the dictionary (ALG-B and MAB), and in cases of conflicting opinion the editors met to discuss and find consensus. In the last quarter of 2023, there were two final review iterations of the draft dictionary with the editorial team. The consensus terms and definitions were compiled into a glossary, which formed the first edition of the *Global Tuberculosis Dictionary.* The glossary was refined to ensure alignment with the Stop TB Partnership’s *Words Matter Language Guide*, and reviewed by two tuberculosis survivors who provided input on the acceptability of the language to the tuberculosis-affected community.^13^

Throughout the document screening and editorial review of the draft dictionary, there were several notable examples of misaligned terms and definitions, which had three common themes: new terms that do not have cohesive understanding in the literature, used in different ways; older terms that do not align with current scientific understanding or currently accepted non-stigmatising language; and definitions that are used inconsistently, in publications from the same organisation, and even from the same year. Extensive discussion within the editorial team revolved around the term tuberculosis itself, which has been used variably, often followed by the word disease (ie, tuberculosis disease), preceded by the word active (ie, active tuberculosis), or both. The term tuberculosis has been used both to refer to the disease (as opposed to infection with *M tuberculosis*), or to the broader field of *M tuberculosis* interactions within the human body. Mimicking the lexicology used in other diseases (eg, HIV infection is the cause of AIDS or SARS-CoV-2 is the cause of COVID-19), our recommendation is to use the term *M tuberculosis* infection to refer to the traditional terms of tuberculosis infection or latent tuberculosis infection, and to use the term tuberculosis to refer to the disease stage caused by *M tuberculosis.* Within the website of the *Global Tuberculosis Dictionary,* we provide a list of many of the terms and definitions that were initially selected for reviewer assessment, and note the variability among them.

The *Global Tuberculosis Dictionary* has been developed through a systematic approach, which was guided by evidence and expert review, to develop a list of terms and definitions to harmonise global tuberculosis-related language. As our understanding of tuberculosis evolves, so too should the terminology and respective definitions. Therefore, new or amended terms and definitions can be proposed through the *Global Tuberculosis Dictionary* website for inclusion in subsequent editions of the dictionary. These suggestions, alongside new terms from literature, will be reviewed on an annual basis by the editorial team, and the dictionary will be subsequently updated. We hope this open-access dictionary represents a useful resource that allows the community of tuberculosis researchers, policy makers, funders, and those affected by tuberculosis to unify around a common understanding of tuberculosis-related vocabulary, with the ultimate goals of improving overall communication and harmonising efforts in tuberculosis control.

## References

[1] Daniel TM. The history of tuberculosis. Respir Med 2006; 100: 1862–70. 16949809 10.1016/j.rmed.2006.08.006

[2] WHO. Global tuberculosis report 2023. 2023. https://iris.who.int/handle/10665/373828 (accessed Feb 10, 2024).

[3] Garrido-Cardenas JA, de Lamo-Sevilla C, Cabezas-Fernández MT, Manzano-Agugliaro F, Martínez-Lirola M. Global tuberculosis research and its future prospects. Tuberculosis (Edinb) 2020; 121: 101917 32279873 10.1016/j.tube.2020.101917

[4] Cobelens F, Suri RK, Helinski M, Accelerating research and development of new vaccines against tuberculosis: a global roadmap. Lancet Infect Dis 2022; 22: e108–20. 35240041 10.1016/S1473-3099(21)00810-0 PMC8884775

[5] Gill CM, Dolan L, Piggott LM, McLaughlin AM. New developments in tuberculosis diagnosis and treatment. Breathe (Sheff) 2022; 18: 210149. 35284018 10.1183/20734735.0149-2021 PMC8908854

[6] MacLean E, Kohli M, Weber SF, Advances in molecular diagnosis of tuberculosis. J Clin Microbiol 2020; 58: e01582–19. 32759357 10.1128/JCM.01582-19 PMC7512154

[7] Ruhwald M, Carmona S, Pai M. Learning from COVID-19 to reimagine tuberculosis diagnosis. Lancet Microbe 2021; 2: e169–70. 33778790 10.1016/S2666-5247(21)00057-4 PMC7979141

[8] WHO. WHO consolidated guidelines on tuberculosis: module 5: management of tuberculosis in children and adolescents. 2022. https://www.who.int/publications-detail-redirect/9789240046764 (accessed Feb 10, 2024). 35404556

[9] Graham SM, Cuevas LE, Jean-Philippe P, Clinical case definitions for classification of intrathoracic tuberculosis in children: an update. Clin Infect Dis 2015; 61 (suppl 3): S179–87. 10.1093/cid/civ581 PMC4583568 26409281

[10] WHO. Consensus meeting report: development of a Target Product Profile (TPP) and a framework for evaluation for a test for predicting progression from tuberculosis infection to active disease. 2017. https://www.who.int/publications/i/item/WHO-HTM-TB-2017.18 (accessed Feb 10, 2024).

[11] Drain PK, Bajema KL, Dowdy D, Incipient and subclinical tuberculosis: a clinical review of early stages and progression of infection. Clin Microbiol Rev 2018; 31: e00021–18. 10.1128/CMR.00021-18 PMC6148193 30021818

[12] Migliori GB, Ong CWM, Petrone L, D’Ambrosio L, Centis R, Goletti D. The definition of tuberculosis infection based on the spectrum of tuberculosis disease. Breathe (Sheff) 2021; 17: 210079. 35035549 10.1183/20734735.0079-2021 PMC8753649

[13] Stop TB Partnership. Words Matter: suggested language and usage for tuberculosis communications. 2022. https://www.stoptb.org/words-matter-language-guide (accessed Feb 10, 2024).

